# Elective surgery for colorectal cancer in the aged: a clinical-economical evaluation.

**DOI:** 10.1038/bjc.1997.394

**Published:** 1997

**Authors:** R. A. Audisio, M. Cazzaniga, C. Robertson, P. Veronesi, B. Andreoni, M. S. Aapro

**Affiliations:** EIO-European Institute of Oncology, Milan, Italy.

## Abstract

A series of 56 consecutive patients, referred for surgery to a specialized institute, had elective laparotomies with various surgical procedures aimed at curing locoregional colorectal cancer. Data defining patient and tumour-related preoperative, operative and postoperative variables, including costs, were collected. The study group was divided into two age groups (< 65 vs > or = 65 years), which were similar in terms of patient- and tumour-related variables. Differences were not statistically significant (Pounds 440; 95% exact CI; Pounds -50; 1800). There is no evidence to suggest that there are any total charge differences in treating the two age groups, as confirmed by the cost analysis.


British Journal of Cancer (1997) 76(3), 382–384
© 1997 Cancer Research Campaign

### Elective surgery for colorectal cancer in the aged: a clinical–economical evaluation

**RA Audisio*, M Cazzaniga, C Robertson, P Veronesi, B Andreoni and MS Aapro***

EIO – European Institute of Oncology, via Ripamonti 435, 20141 Milan, Italy

**Summary** A series of 56 consecutive patients, referred for surgery to a specialized institute, had elective laparotomies with various surgical procedures aimed at curing locoregional colorectal cancer. Data defining patient and tumour-related preoperative, operative and post-operative variables, including costs, were collected. The study group was divided into two age groups (< 65 vs ≥ 65 years), which were similar in terms of patient- and tumour-related variables. Differences were not statistically significant (£440; 95% exact CI; £ –50; 1800). There is no evidence to suggest that there are any total charge differences in treating the two age groups, as confirmed by the cost analysis.

**Keywords:** elderly; colorectal cancer; elective surgery; cost; health economics

Almost 50% of deaths from colorectal cancer occur in patients over 75 years of age (Silverberg et al, 1990) and the median age at diagnosis is around 68.4 years for rectal and 70.5 years for colon cancer (Young et al, 1981), the incidence of colorectal malignancy increasing steadily up to the eight decade (Winawer et al, 1984). A difference in the clinical presentation is being reported (Arnaud et al, 1991) as a consequence of a twofold increase in right-sided cancers, which are responsible for occult bleeding, occlusion and nutritional impairment. Obstructing tumours are significantly more common in patients over 70 years of age (Korenaga et al, 1991), requiring emergency operation; this is associated with a significantly higher incidence of operative deaths at any age (Turunen et al, 1983; Irvin, 1988), but the prevalence of operative morbidity and mortality among the aged on emergency is significantly higher than for younger patients under the same conditions, in both eastern and western series (Korenaga et al, 1991; Mulcahy et al, 1994). When only elective operations are considered, any difference in operative deaths recorded in the two age groups is small, ranging from 4% to 7% (Arnaud et al, 1991; Mulcahy et al, 1994). Kingston et al (1995) recorded a significant increase in the length of hospital stay in the over 75 age group (16 vs 13 days), although post-operative wound infection and leak rates were not significantly different: this was explained by a more detailed time-consuming preoperative evaluation. Finally, survival is better for younger patients, but this difference loses statistical significance when survival curves plotted for patients undergoing potentially curative surgery and malignant deaths are considered (Mulcahy et al, 1994). This stimulated our interest in assessing the difference in the economical burden when delivering radical surgery to the aged colorectal cancer patient vs younger ones, in order to address a common clinical problem. The advantage of doing such a study at the EIO, Milan, is that we can provide all information about costs in a European setting. In these times of health economic evaluation and of hospital purchasing, it is essential to know whether the allocation of the financial resources is equitable. A higher cost of treatment in the elderly compared with the young with the same disease could implicate, if budgetary reasons prevail, an ethical problem as some could take cost as a reason not to treat.

### PATIENTS AND METHODS

The consecutive series of 56 stage I–III colorectal cancer subjects, all of whom were surgically treated for cure at the European Institute of Oncology, Milan, from its opening in May 1994 to June 1996, are entered into the present study. There is no selection bias of patients once they have been referred to this institute. Several variables were collected, including patient- and tumour-related ones (Table 1), perioperative data (Table 2) and costs (Table 3). Costs are expressed in pounds sterling and define (a) preoperative real costs, which include diagnostic and staging packages, tests and investigations used to assess operability as well as specific cardiologic investigations when required; (b) operative real costs which include computing, surgical and anaesthesiological equipment but exclude the costs of the staff; (c) post-operative real costs, which include the real costs of daily hospital or ICU stay times number of days of admittance, drugs and blood units delivered to each patient, post-operative tests and investigations, reinterventions and the staff real cost, defined as the daily salary of each staff member multiplied by the number of days spent in the unit; and (d) total charge to the patient from a private non-profit Italian institution, as the EIO is, representing the bill the patient is asked to pay when leaving the hospital. A cut-off point of 65 years was used to dichotomize the series into two age groups to be compared. The differences in median costs between the two groups were tested according to the Wilcoxon rank sum test and confidence intervals provided by the Hodges–Lehmann estimate; Fisher's exact test was used for testing equality of proportions.

*Received 8 July 1996*
*Revised 24 January 1997*
*Accepted 30 January 1997*

*Correspondence to:* RA Audisio, General Surgery 1, EIO – European Institute of Oncology, via Ripamonti 435, 20141 Milan, Italy

*Member of GIOGer. (Italian Group for Geriatric Oncology)

**Table 1** Patient and tumour-related variables

| | < 65 years | ≥ 65 years |
|---|---|---|
| Number of patients | 26 | 30 |
| Median age (years) | 57 | 86 |
| Range (years) | 47–63 | 65–86 |
| Gender | | |
| Male/Female | 16/10 | 17/13 |
| Associated diseases | | |
| No | 6 | 7 |
| Yes | 20 | 23 |
| Tumour site | | |
| Right colon | 1 | 6 |
| Transverse | 2 | 0 |
| Left | 9 | 11 |
| Rectum | 14 | 13 |
| Grading | | |
| 1 | 8 | 9 |
| 2 | 17 | 18 |
| 3 | 1 | 3 |
| Surgery | | |
| Right colectomy | 1 | 6 |
| Transverse colectomy | 2 | 0 |
| Left colectomy | 9 | 11 |
| Anterior resection | 9 | 8 |
| APR (Miles) | 5 | 5 |

**Table 2** Operative variables

| | < 65 years | ≥ 65 years |
|---|---|---|
| Median operation hours | 3.75 | 3.0 |
| Range | 1.5–6.5 | 2.0–5.0 |
| Median number blood units | 0 | 0 |
| Range | 0–5.0 | 0–47.0 |
| Median ICU stay (days) | 0 | 0 |
| Range | 0–3 | 0–65 |
| Median post-operative stay (days) | 10 | 10 |
| Range | 7–47 | 8–88 |
| Thirty-day morbidity | | |
| No | 20 | 17 |
| Yes | 6 | 13 |
| Thirty-day mortality | | |
| No | 26 | 30 |
| Yes | 0 | 0 |

**Table 3** Costs and charges in sterling

| | < 65 years | ≥ 65 years |
|---|---|---|
| Preoperative investigations | 400 | 400 |
| Range | 400–480 | 400–520 |
| Operative real costs[a] | 1040 | 1040 |
| Range | 880–1040 | 800–1040 |
| Post-operative real costs[b] | 6280 | 6680 |
| Range | 4440–32 040 | 5040–23 260 |
| Median total charges | 7720 | 8160 |
| Range | 5880–33 480 | 6480–24 540 |

[a]Staff excluded; [b]Staff included.

### RESULTS

The patients entered into the study represent all those referred for surgery to our centre. The two groups were similar in terms of clinicopathological variables (Table 1). A slight increased prevalence of right-sided colon cancers was detected among patients ≥ 65 years, which was responsible for 20% of right colectomies vs 3.8% in the younger age group, ($P = 0.11$).

The overall length of the operation ranged from 1 h 30 min to 6 h 30 min, with a median time of 3 h 30 min (Table 2). Special care in avoiding uncontrolled bleeding is our policy, and most patients had no transfusion at all; five patients aged < 65 required homologous blood, compared with nine patients in the ≥ 65 years age group. Patients were sent to the ICU on the basis of a multiparametric scoring system that took account of preoperative assessment, length of operation and blood loss, number of blood units replaced and peroperative surgical and anaesthesiological complications. Median ICU stay was 0 days for both age groups, but 6 out of 20 patients < 65 had more than 1 day in the ICU vs 12 out of 30 among the ≥ 65-year-old group. The difference in operative (30 days) morbidity was statistically not significant, and none of the groups showed any post-operative mortality.

When costs are analysed (Table 3), the preoperative investigational package is standardized for all colorectal cancer patients, no matter the age group; thus minor variations are to be ascribed to Holter monitoring and pulmonary function testing occasionally performed in the elderly at the cardiologist's request. Operative real costs are equivalent in both groups because of the use of a standard operative technique, and consequently there are similar expenses for the operative equipment. A moderate, but not statistically significant ($P = 0.07$), increase in post-operative real costs for the ≥ 65 years old subjects (the median is £400 greater) is to be ascribed to a more intensive post-operative monitoring. The median total charge to the patient is also similar in both age groups. There is no evidence to suggest that there are major differences in treating the two age groups, as confirmed by the cost analysis. The difference in the median costs is £440, with a 95.2% exact confidence interval for the differences in the total costs between patients in the two groups of £ (–50; 1800). Similar conclusions are reached if the study population is divided around the median age of 69 years.

The median age at diagnosis for colon cancer is 69, and using this as a cut-off point to give two groups, one with age less than or equal to 69 and the other aged 70 or over, yielded similar results. Specifically, the difference of the median costs is £400, with a 95.2% exact confidence interval for the differences in the total costs between patients in the two groups of –£120 and £1750.

### DISCUSSION

Surgery is the first choice treatment for colorectal cancer patients, the most prevalent cancerous disease among elderly subjects: this age group does not show an increased operative morbidity and mortality when operated under elective conditions, and is almost four times more frequently treated than in the 1940s (Lea et al, 1982). This study has addressed a group of patients referred to a tertiary centre for elective surgery. The conclusion therefore cannot apply to a non-selected population with severe co-morbidity.

Admittedly, if we consider the £400 median difference in cost per patient and we multiply it by the number of elderly patients treated in this institute, then the total cost could be £12 000 more than if all patients were less than 65 years old compares with a total budget of £500 000 for treating 56 patients. This represents a 2–3% increase on the total budget. Even at the most extreme confidence limit, the excess costs of treating elderly patients are less than 10% of the total budget. These are small increments on a

© Cancer Research Campaign 1997

large sum of money, and it is a political decision to determine how to allocate a country's budget: to the military, explorations, tobacco subsidies, industry or health.

Financial issues intrude on medicine from all fronts, and cost analysis of common conditions is required to address clinical practice. A commonly proposed reason for not treating elderly patients is that one should consider their active life expectancy (Katz et al, 1983), which obviously increases apparent cost in health economic terms. This is a political decision that a physician should not have to face (Sulmasy, 1992). It has been demonstrated that strategies to improve the prognosis of colorectal cancer should not exclude the older patients (Mulcahy et al, 1994), and our small prospective series contributes to this conclusion.

This is a small series of data, but one that deals with an important area. It is correct to conclude that one of the major sources of cost difference between the two groups is likely to be serious adverse events requiring expensive treatment. One would expect that this is more likely to occur in the older group. In our study there were two adverse events in each group, all of which had very high costs.

### REFERENCES

Arnaud JP, Schloegel M, Ollier JC and Adloff M (1991) Colorectal cancer in patients over 80 years of age. *Dis Colon Rectum* **34**: 896–898

Irvin TT (1988) Prognosis of colorectal cancer in the elderly. *Br J Surg* **75**: 419–421

Katz S, Branch LG, Branson MH, Papsidero JA, Beck JC and Greer DS (1983) Active life expectancy. *N Engl J Med* **309**: 1218–1224

Kingston RD, Jeacock J, Walsh S and Keeling F (1995) The outcome of surgery for colorectal cancer in the elderly: a 12-year review from the Trafford Database. *Eur J Surg Oncol* **21**: 514–516

Korenaga D, Ueo H, Mochida K, Kusumoto T, Baba H, Tamura S, Moriguchi S and Sugimachi K (1991) Prognostic factors in Japanese patients with colorectal cancer: the significance of large bowel obstruction; univariate analyses. *J Surg Oncol* **47**: 188–192

Lea JW, Covington K, McSwain B and Scott HW (1982) Surgical experience with carcinoma of the colon and rectum. *Ann Surg* **195**: 600–607

Mulcahy HE, Patchett SE, Daly L and O'Donoghue DP (1994) Prognosis of elderly patients with large bowel cancer. *Br J Surg* **81**: 736–738

Silverberg E, Boring CC and Squires TS (1990) Cancer Statistics, 1990. *Ca Cancer J Clin* **40**: 9–26

Sulmasy DP (1992) Physicians, cost control, and ethics. *Ann Intern Med* **116**: 920–926

Turunen MJ and Peltokallio P (1983) Surgical results in 657 patients with colo-rectal cancer. *Dis Colon Rectum* **26**: 606–612

Winawer SJ, Miller DG and Sherlock P (1984) Risk and screening for colorectal cancer. *Adv Int Med* **30**: 471–496

Young JL, Percy CL, Asire AJ, Berg JW, Cusano MM, Gloeckler LA, Horm JW, Lourie WI Jr, Pollack ES and Shambaugh EM (1981) Cancer incidence and mortality in the United States, 1973–77. *Natl Cancer Inst Monogr* **57**: 1–187

© Cancer Research Campaign 1997


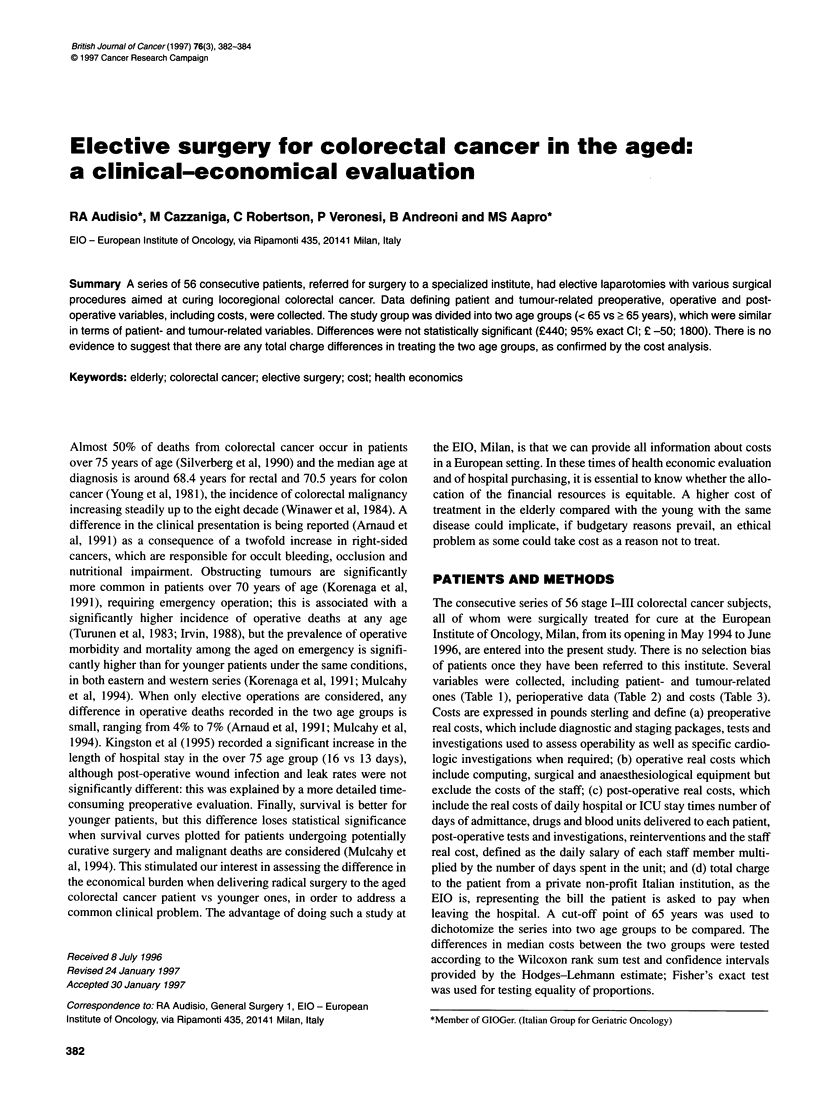

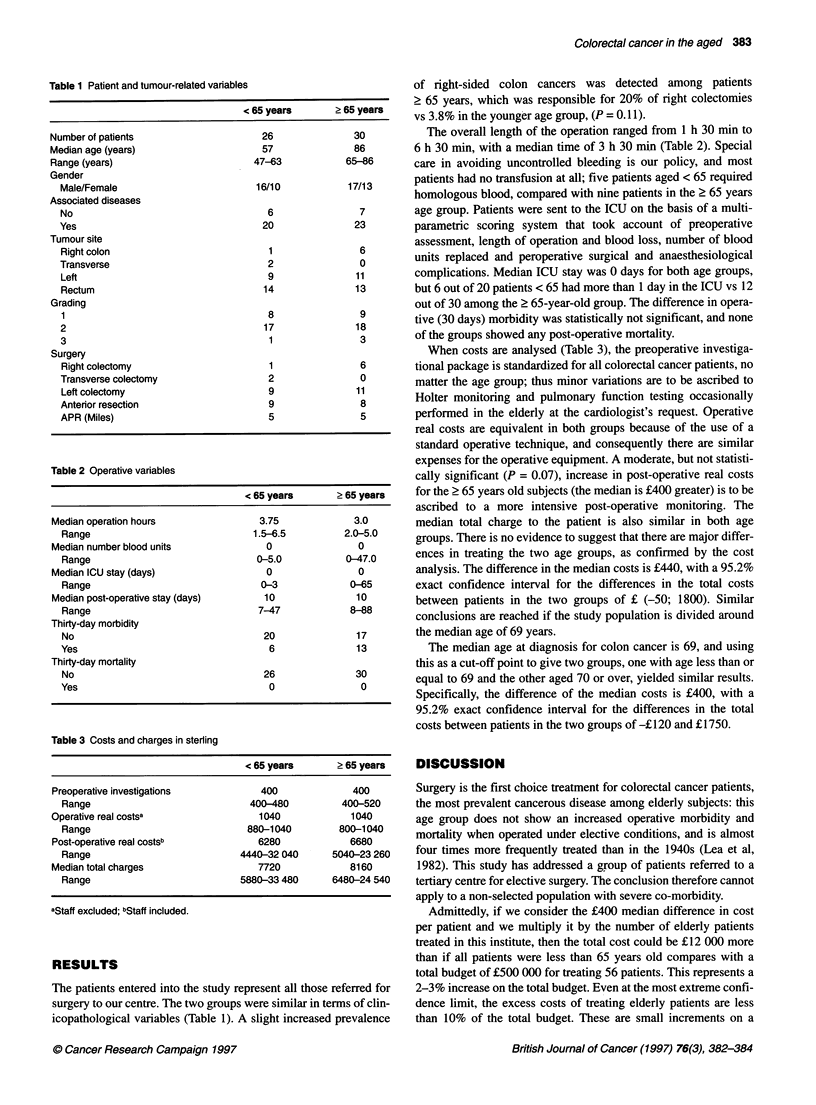

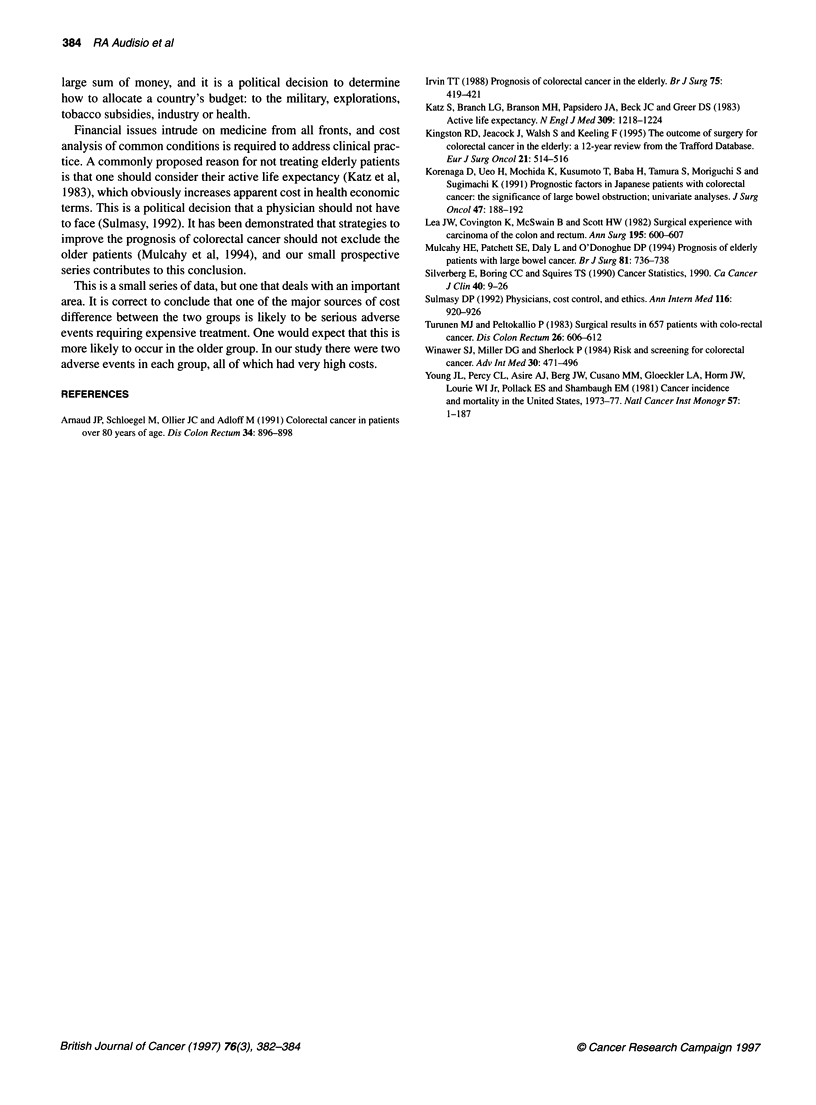


## References

[OCR_00318] Arnaud J. P., Schloegel M., Ollier J. C., Adloff M. (1991). Colorectal cancer in patients over 80 years of age.. Dis Colon Rectum.

[OCR_00322] Irvin T. T. (1988). Prognosis of colorectal cancer in the elderly.. Br J Surg.

[OCR_00326] Katz S., Branch L. G., Branson M. H., Papsidero J. A., Beck J. C., Greer D. S. (1983). Active life expectancy.. N Engl J Med.

[OCR_00330] Kingston R. D., Jeacock J., Walsh S., Keeling F. (1995). The outcome of surgery for colorectal cancer in the elderly: a 12-year review from the Trafford Database.. Eur J Surg Oncol.

[OCR_00335] Korenaga D., Ueo H., Mochida K., Kusumoto T., Baba H., Tamura S., Moriguchi S., Sugimachi K. (1991). Prognostic factors in Japanese patients with colorectal cancer: the significance of large bowel obstruction--univariate and multivariate analyses.. J Surg Oncol.

[OCR_00342] Lea J. W., Covington K., McSwain B., Scott H. W. (1982). Surgical experience with carcinoma of the colon and rectum.. Ann Surg.

[OCR_00346] Mulcahy H. E., Patchett S. E., Daly L., O'Donoghue D. P. (1994). Prognosis of elderly patients with large bowel cancer.. Br J Surg.

[OCR_00350] Silverberg E., Boring C. C., Squires T. S. (1990). Cancer statistics, 1990.. CA Cancer J Clin.

[OCR_00354] Sulmasy D. P. (1992). Physicians, cost control, and ethics.. Ann Intern Med.

[OCR_00358] Turunen M. J., Peltokallio P. (1983). Surgical results in 657 patients with colorectal cancer.. Dis Colon Rectum.

[OCR_00362] Winawer S. J., Miller D. G., Sherlock P. (1984). Risk and screening for colorectal cancer.. Adv Intern Med.

[OCR_00366] Young J. L., Percy C. L., Asire A. J., Berg J. W., Cusano M. M., Gloeckler L. A., Horm J. W., Lourie W. I., Pollack E. S., Shambaugh E. M. (1981). Cancer incidence and mortality in the United States, 1973-77.. Natl Cancer Inst Monogr.

